# The role of meditation and mindfulness in the management of polycystic ovary syndrome: a scoping review

**DOI:** 10.3389/fendo.2024.1295705

**Published:** 2024-05-16

**Authors:** Vibhuti Rao, Alexia Pena, Annie James, Aashish Phadke, Jahnavi Grover, Ella Blendis, Nidhi Choudhary, Punith Kampegowda

**Affiliations:** ^1^ NICM Health Research Institute, Western Sydney University, Penrith, NSW, Australia; ^2^ Discipline of Paediatrics, The University of Adelaide and Robinson Research Institute, Adelaide, SA, Australia; ^3^ School of Social Sciences, Christ University, Bengaluru, India; ^4^ Division of Endocrine and Metabolic Disorders, Kasturba Health Society’s Medical Research Centre, Mumbai, India; ^5^ Medical School, Western Sydney University, Penrith, NSW, Australia; ^6^ Medical School, University of Birmingham, Birmingham, United Kingdom; ^7^ Vivekananda Yoga University, Norwalk, CA, United States; ^8^ Institute of Applied Health Research, University of Birmingham, Birmingham, United Kingdom; ^9^ Queen Elizabeth Hospital Birmingham, University Hospitals Birmingham NHS Foundation Trust, Birmingham, United Kingdom

**Keywords:** polycystic ovary syndrome, PCOS, meditation, mindfulness, yoga, lifestyle, Dhyana

## Abstract

Polycystic Ovary Syndrome (PCOS) presents multifaceted challenges affecting women’s reproductive, metabolic, and psychological systems, consequently impacting their psychological and emotional well-being. The utilization of meditation and mindfulness interventions (MMIs) is found to be increasing for the management of PCOS. This scoping review systematically explored the current literature to identify the type and application of MMIs for PCOS management. A systematic search of literature was conducted using CINAHL, PsycINFO, Scopus, MEDLINE, and PubMed databases for identifying studies conducted on the usage of MMIs in women diagnosed with PCOS, irrespective of age. The comprehensive search identified 14 trials (comprising 17 citations) meeting inclusion criteria, involving 723 participants across various age groups. Among these, nine were randomized controlled trials (RCTs), while the remaining comprised non-RCTs. Several types of MMIs, including *Rajayoga* of *Brahmakumaris*, *Yoga Nidra*, OM cyclic meditation, unspecified forms of meditation, mindfulness-based stress reduction programs, mindful yoga, and mindfulness-based activities, were used. Outcomes were predominantly assessed in psychological domains (n=11), followed by anthropometric (n=9), quality of life (n=7), and metabolic metrics (n=7). The review findings suggest the integration of meditation with conventional treatment modalities. Preliminary data indicate that MMIs have the potential to improve psychosocial well-being and quality of life among PCOS-affected women. However, adequately powered studies with extended follow-up periods are required to investigate the mechanisms and therapeutic efficacy of MMIs, particularly concerning reproductive outcomes and weight management. Furthermore, diligent monitoring and reporting of adverse events and adherence are essential for a comprehensive understanding of MMI utilization in PCOS management.

## Introduction

Polycystic Ovary Syndrome (PCOS) is a chronic disorder prevalent among women of all ages with a global prevalence of about 10 to 13% (1). PCOS can manifest with various symptoms across different life stages, including reproductive, dermatological, metabolic, and psycho-socio-sexual problems (2). Despite this multifaceted nature, the psychological, emotional, and social aspects associated with PCOS often receive inadequate attention in clinical practice, leaving many adolescents and women dissatisfied with the prevailing standard of care (3–6). Consequently, women with PCOS seek personalised healthcare that can address their specific health concerns, especially focusing on psychological and emotional well-being and resilience (7–10).

Several studies have indicated an increasing interest among those affected by PCOS in using various traditional, complementary, and integrative medicine (TCIM), such as yoga, meditation, and mindfulness-based therapies gaining popularity in the last few years (11–13). In a cross-sectional survey, it was reported that approximately 80% of ethnic Indian women worldwide diagnosed with PCOS incorporate some form of TCIM into their management regimen, with yoga being the most commonly adopted practice (12). Therefore, it is essential to explore and understand these practices and their potential implications in mitigating PCOS symptoms.

Meditation, originating from the yoga tradition as the seventh limb termed *Dhyana*, has rich historical origins spanning over 6,000 years (14). Broadly, meditation involves a diverse range of self-taught, self-focused and self-regulatory practices aimed at auto-regulation of neurobehavioral energy, logical relaxation, and achieving a state of ‘unitive being’, with a primary focus on mental training and awareness (15, 16). Meditation encompasses a range of ancient practices originating from Buddhist, Chinese, and Vedic traditions (17). Lutz categorised meditation into two distinct types: focused attention meditation, characterised by an intentional and prolonged concentration on a selected object, and open monitoring meditation, characterised by non-reactive observation of moment-to-moment experiential content (18). Travis and Shear (2010) introduced a third category of meditation practice, termed automatic self-transcending, to help determine its mechanisms or clinical effects encompassing techniques explicitly designed to surpass their own cognitive and operational activities (19). Each category exhibits unique neural correlates, with automatic self-transcending associated with alpha-1 activity, focused attention with beta/gamma activity, and open monitoring with theta activity, drawing upon various traditions including Tibetan Buddhist, Buddhist, and Chinese (19).

Mindfulness, deeply rooted in Buddhist culture, is the most prevalent form of meditation in contemporary research. John Kabat-Zinn defines mindfulness as “the awareness that emerges through paying attention on purpose, in the present moment, and nonjudgmentally to the unfolding of experience moment to moment” (20). Mindfulness practices, whether formal (e.g., breathing, sitting, walking, body scan) or informal (e.g., mindfulness in daily activities), aim to cultivate a state of mindful awareness (21). Several standardised interventions, such as Mindfulness-based Stress Reduction (MBSR) developed by Jon Kabat-Zinn in 1979, and Mindfulness-based Cognitive Therapy (MBCT) developed by Segal, Teasdale and Williams, along with non-standardized mindfulness programs, such as mindful breathing and body scan (22, 23), are of current interest (24–26).

Meditation practices contribute to adaptive cognitive and affective functioning, enhancing positive traits, behaviours, and overall well-being (27). By promoting awareness and regulating attention, meditation aims to relieve physical, mental and emotional distress (28). A comprehensive review of 191 systematic and meta-analytic reviews highlights the versatility of meditation in addressing various health conditions, including anxiety, depression, stress, quality of life (QoL), fatigue, weight management, sleep quality, daily activities, and behavioural changes (29). Mindfulness-based interventions have demonstrated efficacy in managing pain, and negative emotions, cultivating self-compassion, and enhancing mindfulness, social connections, and positivity (30).

Employing additional strategies beyond the symptomatic treatment of PCOS becomes increasingly important in the holistic management of women with PCOS. Moreover, cost-effective strategies capable of alleviating psychological burdens, promoting healthy behavioural changes, and enhancing overall quality of life are increasingly warranted. Previous systematic reviews have reported inconsistent findings regarding the efficacy of yoga-based meditation and mindfulness in managing PCOS symptoms (11, 31). Meditation was not assessed independently in these reviews, and recent studies have since been published. Therefore, the objective is to provide an updated summary of the evidence pertaining to meditation/mindfulness interventions (MMIs) and their therapeutic potential in optimising PCOS management.

## Methods

The methods employed in this study adhered to a predetermined protocol. The search strategy and methods for extracting data were congruent with the Preferred Reporting Items for Systematic Reviews and Meta-Analyses extension for Scoping Reviews (PRISMA-ScR) checklist. Extensive literature searches were conducted across multiple databases including Medline OVID, Scopus, PubMed, PsychInfo, and CINAHL, spanning from inception to March 2023 for identifying current evidence on MMIs in PCOS. The keywords and search strategies used in Medline are described in **Supplementary Table 1**, with similar search strategy approaches applied to other databases. Original experimental studies published in English on meditation/mindfulness alone or in combination with other treatments in women diagnosed with PCOS using research designs such as randomised controlled trials (RCTs), quasi-experimental, single-arm, and pre-post were included. No restrictions were imposed regarding participants’ age, PCOS diagnosis criteria or the type or duration of the MMIs used. Excluded from consideration were reviews, case studies/series, qualitative studies, and studies utilising alternative research methodologies. Citations were extracted and duplicates were removed using Endnote (32).

Covidence (33) was used to manage the screening process. VR, JG and APD conducted the abstract and full-text screening in duplication. Any discrepancies that arose from the two screening processes were resolved by discussion and consensus, with consultation among all authors where needed. Data extraction tables were initially developed by one author (VR) and subsequently refined based on feedback from two authors (APD, NC). Data extraction from eligible studies was conducted independently by one author (VR) using Microsoft Excel (version 16.68) and subsequently verified for accuracy and completeness by another author (AJ). Extracted data included study design, participant demographics and settings, intervention characteristics, and any reported adverse events and outcomes related to PCOS features. A narrative descriptive summary of the current evidence was constructed employing the Population, Intervention, Comparison, and Outcome (PICO) method.

## Results

### Study selection

**Figure 1:**
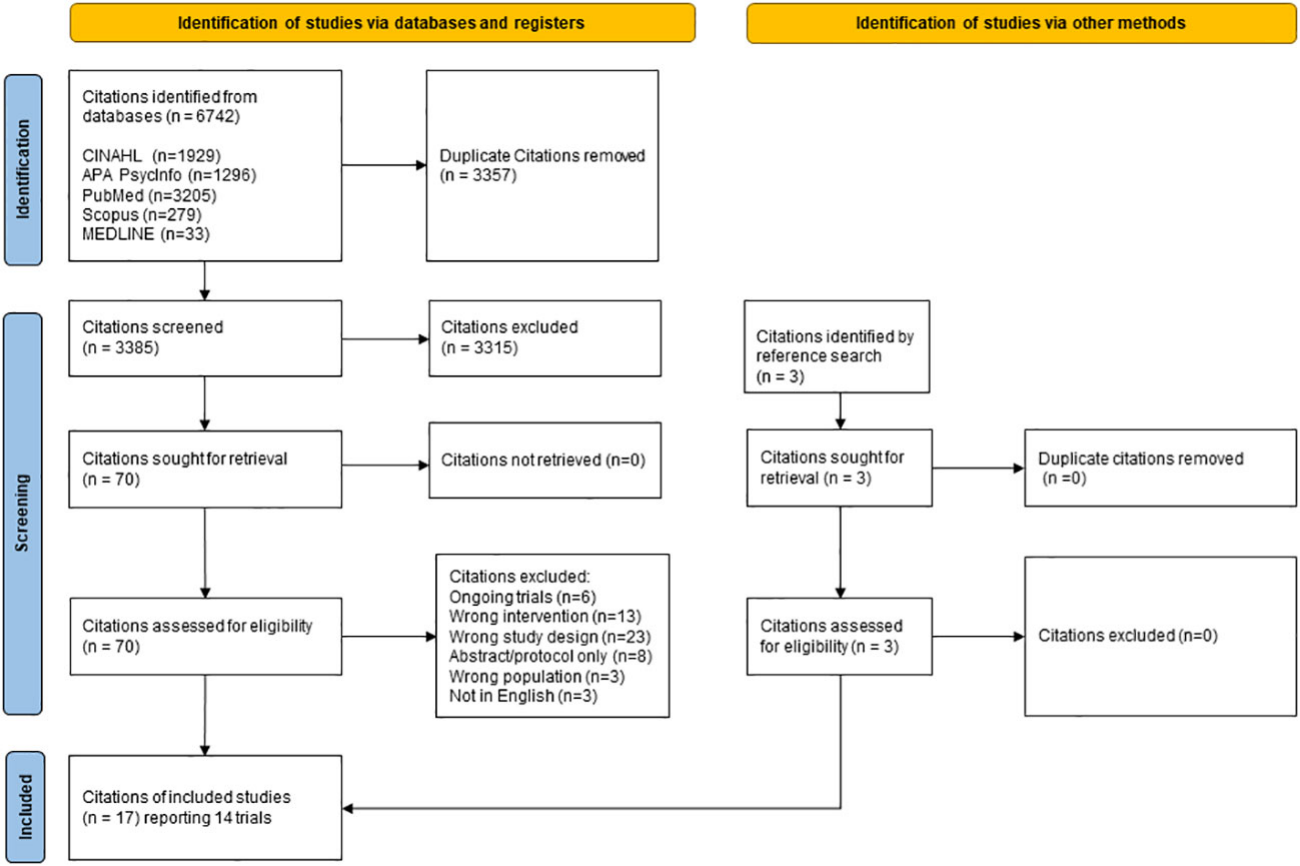
Legend: Flow chart of the study selection process.

The search strategy yielded 6742 records, of which 3385 independent studies were eligible for title and abstract screening after the removal of duplicates. Finally, 70 citations were retrieved for full-text screening, out of which 17 citations representing 14 trials (one trial having four citations) satisfied the inclusion criteria and were included in the analysis. The article selection process is visually presented in the PRISMA flow chart (**Figure 1**).

### Study Characteristics

Among the 14 trials, nine trials were RCTs (12 citations) (34–45), four were single-arm trials (46–49) and one was a quasi-experimental trial (50). The distribution of trials across geographical locations included six trials (9 citations) conducted in India (34–37, 39, 44, 46, 47, 49), four in the USA (40, 42, 45, 48), and one each in China (41), Iran (50), Greece (38) and Slovenia (43). Refer to **Table 1** for a summary of available evidence.

**Table 1:** Summary of trials investigating the role of meditation and mindfulness in PCOS.

Study details (Author, year, country)	Study design	Participants (Number, age, diagnosis)	Intervention details related to meditation/mindfulness	Duration, frequency, and total duration of the intervention	Control/comparison	Outcomes with significant findings related to intervention
Nidhi et al., 2012, India (34–37)	RCT	90, 15–18 years, Rotterdam criteria	*Mantra* meditation included in a one-hour yoga therapy session	One hour Daily for 12 weeks	Matched physical activity	↓ mFG score, LH, LH/FSH, AMH, Total testosterone, FI, FBG, HOMA-IR, TC, TG, LDL, VLDL, TC/HDL, trait anxiety (STAI) ↑ HDL, menstrual cycle frequency and quality of life (PCOSQ) ↔ weight, BMI, WC, WHR, FSH, Prolactin, and state anxiety. Adverse events not reported.
Stefanaki 2014, Greece (38)	Pilot RCT	38, 15-40 years, Rotterdam criteria	MBSR program (including mindfulness breathing exercises and diaphragmatic breathing exercises) using an audio compact disc	30 minutes, daily for 8 weeks	No intervention	↓ depressive, anxiety, and stress symptoms (DASS 21), salivary cortisol concentrations, ↑ quality of life scores (PCOSQ), total life satisfaction and general satisfaction, (Routine-daily life questionnaire). ↔ stress (PSS-14), relationship and job satisfaction (Routine-daily life questionnaire) No adverse event occurred.
Sode 2017, India (49)	Single arm pre-post trial	30, 25-35 years, Criteria not specified	Non-specified meditation included in a one-hour yoga therapy session	Unspecified duration and frequency for 60 days,	NA	↓ depression (BDI-II) Adverse events not reported.
Raja Khan 2017, USA (45)	Pilot RCT	86, age ≥18 years with BMI ≥ 25 kg/m^2^, NIH criteria	Modified MBSR program (with shorter duration of home practice) .	2.5 hours of weekly sessions and 6 hours of retreat sessions with 25-30 minutes of home practice for 8 weeks	Health education guidelines on diet, lifestyle, and stress management	↑ mindfulness state (TMS), quality of life (SF-36), ↓ perceived stress (PSS-10), overall psychological distress, depression and anxiety on BSI-18, fasting glucose, Improved sleep (PROMIS Sleep-Related Impairment T-score), PANAS Negative and positive Affect Scores, ↔ weight, BMI, WC, BP, fasting insulin, HOMA-IR, Lipid profile, HbA1c, Salivary cortisol and HsCRP. No adverse event occurred.
Rao 2018, India (39)	RCT	64, 18 to 40 years, Rotterdam criteria	*Mantra* meditation included in a one-hour yoga therapy session	One-hour session 5 times per week for 12 weeks	Whole system Ayurvedic treatments	↓ HbA1C, ovary mass, stress, anxiety and depression (HADS), ↔ weight, Prolactin, FSH, LH, AMH, TT, No adverse events occurred.
Vanitha 2018, India (46)	Single arm pre-post	40, 18 and 35 years, Rotterdam criteria	*Yoga Nidra* was described by Swami Satyananda Saraswati from Munger, Bihar.	40 minutes, daily once for 12 weeks	NA	↓ resting heart rate, SBP, DBP, Weight, BMI, WHR, Adverse events not reported
Verma 2019, India (47)	Single arm pre-post	30, 20-35 years Newly diagnosed Rotterdam criteria	*Mantra* meditation included in a one-hour integrated yoga therapy session	60 minutes, 6 days per week for 12 weeks	NA	↓ BMI Improved heart rate variability (HRV) Adverse events not reported
Patel 2020, USA (40)	RCT	31, 22-43 years, with BMI ≥ 20 kg/m^2,^ Rotterdam criteria	Mindful yoga meditation is included in a one-hour yoga therapy session	60 minutes, 3 times per week for 12 weeks	No intervention	↓ free testosterone, DHEA, anxiety (BAI), depression (BDI-II), acne improved menstrual frequency ↔ DHEA-S, A4, mFG score, FBG, FI, HOMA-IR, BMI and WHR Adverse events not reported
Missel 2021, USA (48)	Single-arm prospective pilot study	29, 21–40 years with BMI ≥ 25 kg/m^2^ Criteria not specified	Mindfulness activities along with a very low carbohydrate dietary program	10-30 minutes weekly 16 weeks	NA	↓ weight, BMI, HbA1C, LDL, calorie intake, food craving to starch and sweets (FCI), Fatigue (PROMIS) ↑ SHBG, quality of life (PCOSQ), Mindful eating, global physical and mental health (PROMIS) ↔ TG, HDL, Glucose, Insulin, Testosterone, HOMA-IR, sleep (PROMIS) Adverse events occurred and were reported.
Yin 2021, China (41)	Pilot RCT	18, 18–35 years Rotterdam criteria	Meditation included in an Integrative body-mind-spirit (I-BMS) program	3 hours weekly session for 6 weeks	No intervention	↓ anxiety (BAI), depression (BDI-II), testosterone, TG, TG/HDL-C, BMI. ↑ total quality of life (PCOSQ) ↔ FBS, TC, HDL-C, LDL-C. Adverse events are not reported.
Young 2022, USA (42)	Pilot RCT	51, 14–23 years, Criteria not specified	Mindfulness-based healthy lifestyle self-management intervention (the PCOS Kind Mind Program)	60 to 75 minutes weekly for 5 weeks	No intervention	↑ nutrition self-efficacy (DIET-SE) and physical activity self-efficacy and strategies (PACE) ↔ self-esteem (RSE), depression, anxiety and stress (DASS-21), and mindfulness (CAMM). Adverse events not reported
Dema 2023, Slovenia (43)	RCT	42, Mean age 40.1 (SD = 7.3) years Rotterdam criteria	MBSR program	120 minutes weekly for 8 weeks	No intervention	Positive changes in DNA Methylation of genes ↓ weight, BMI, WC, Plasma glucose at 0 minutes and 120 minutes, anxiety (BAI), perceived stress score (PSS-20) ↑ Mindfulness-like traits (FFMQ), Quality of life (SF-36 questionnaire) on general health, pain, and emotional health. ↔ depression (BDI-II), Mean arterial blood pressure, heart rate, total cholesterol, HDL, and LDL, TG. Adverse events not reported
Salajegheh 2023, Iran (50)	Quasi-experimental	60, 15-45 years, Rotterdam criteria	MBSR program	90 minutes twice a week for 4 weeks	No intervention	↓ PCOS-related worries (Worry questionnaire). Adverse events not reported.
Renuka Jakhar, 2023, India (44)	RCT	114, 18-45 years, Rotterdam criteria	*Rajayoga* meditation described by *Brahmakumaris*	45 minutes daily for 12 weeks	*Rajayoga* meditation for 30 minutes once a week weekly	↑ Quality of life in all the domains (PCOSQ) Adverse events not reported

NA, Not Applicable.

### Participants

The collective sample size across the 14 trials comprised 723 participants, with individual trial sample sizes ranging from 18 to 114 participants. Age ranges varied across trials, with one trial specifically involving individuals aged 15 to 18 years (34–37). Three trials involved both adolescent and adult participants, with one study involving individuals aged 15 to 40 years (38), another involving individuals aged 14 to 23 years (42), and a third study involving individuals aged 15 to 45 years (50). The remaining ten trials focused on participants aged 18 and above. Diagnostic criteria for PCOS varied across trials, with one trial using the National Institutes of Health (NIH) criteria (51), three trials not specifying the diagnosis criteria (42, 48, 49), and the remaining 10 trials using the Rotterdam criteria.

### Interventions

Several meditation practices, such as Rajayoga meditation, described by *Brahmakumaris* (44), and *Yoga Nidra* (46), were used. Three trials (6 citations) used OM meditation in holistic yoga therapy, including other components of yoga, such as *asana* and *pranayama* (breathing) practices (34–37, 39, 47). Two trials included unspecified forms of meditation (41, 49). Four studies used the MBSR program (38, 43, 50, 51). One trial included mindfulness-based activities, such as mindful eating (48), and another trial employed a mindfulness-based healthy lifestyle self-management program (PCOS kind mind) (42). One trial used mindful yoga (40). Intervention durations ranged from 10 minutes to 2.5 hours per session, spanning study durations of 4 to 16 weeks. Most interventions were administered by trained therapists, with one study comparing different durations of meditation practices (44). Adherence was reported in five trials (39, 41, 42, 48, 51).

### Control/comparisons

Among the 14 trials, four were single-arm trials and thus lacked a control intervention (46–49). Four trials used active interventions (34–37, 39, 44, 51), while the remaining six used control groups without active interventions.

### Outcomes

The studies investigated various PCOS-related outcomes, with psychological parameters being the most commonly examined (n=11/14), followed by anthropometric (n=9/14), metabolic (n=7/14) and QoL outcomes (n=7/14). Reproductive outcomes were reported in four trials. Three trials examined the impact of mindfulness-based interventions on participants’ mindfulness. Three trials investigated heart rate variability, two trials studied sleep, two trials studied diet and physical activity-related parameters, and one study explored fatigue.

Various psychological outcomes, including anxiety, depression, stress, PCOS-related worries, and overall psychological distress, were examined. Several subjective questionnaires/tools were used to evaluate these psychological outcomes among participants. The used questionnaires/tools included Beck’s Depression Inventory-II (40, 41, 43, 49), The Depression, Anxiety and Stress Scale - 21 Items (38, 42), Hospital Anxiety and Depression Scale (39), Beck Anxiety Inventory (40, 41, 43), The State-Trait Anxiety Inventory (36), Brief Symptoms Inventory-18 (45), and The Perceived Stress Scale (38, 43, 45). Most trials reported improvement in psychological parameters, except for one 5-week study that indicated the need for an extended follow-up assessment to comprehensively evaluate psychological parameters (42).

The effects on anthropometric parameters were varied. Five of the nine trials showed improvement in anthropometric parameters: weight (43, 46, 48), BMI (41, 43, 46–48), waist-to-hip ratio (46) and waist circumference (43). However, four trials reported no significant improvement in these parameters (37, 39, 40, 51). None of the four trials reporting on reproductive outcomes assessed pregnancy and related outcomes. Among these, two trials reported an improved frequency of the menstrual cycle (34, 40), while one reported a decrease in ovarian mass (39). Two trials reported clinical hyperandrogenism evaluated by the modified Ferriman-Gallwey (mFg) score, with a significant decrease in one trial (34) and no significant changes in another trial (40). Two trials reported decreased biochemical hyperandrogenism – total testosterone (34, 41), free testosterone (40), and dehydroepiandrosterone (40). One trial reported increased levels of sex hormone-binding globulin (48).

Improvement in QoL of life was reported in seven trials (38, 41, 43–45, 48); the Polycystic Ovary Syndrome Quality of Life (PCOSQ) scale and Short Form Survey (SF-36) were used to assess the QoL among the participants. Three trials reported improvement in mindfulness-related traits (43, 48, 51) using the Toronto Mindfulness Scale and Five Facet Mindfulness Questionnaire for evaluating mindfulness. Two trials studied the effect of meditation on heart rate variability (46, 47). One trial studied changes in DNA methylation (43), self-efficacy regarding diet, physical activity, sleep and behavioural changes (42), general and physical health (48), and overall life satisfaction (38). The mindful eating trial observed less calorie intake and reduced cravings for sweets and carbohydrates as improvements associated with mindful eating.

Whilst most trials (n=10) did not mention the occurrence or non-occurrence of adverse events during the study period, three trials specified the absence of adverse events (38, 39, 51). One trial reported the incidence of adverse events, including kidney stone development, continuous muscle cramping and blurry vision (48). It is important to note that this trial was predominantly focused on dietary-dominant mindfulness interventions and the causal relationship between these adverse events and mindfulness remains uncertain.

In sum, psychological outcomes were evaluated using various standardised tools, with most trials reporting improvements in psychological parameters. Improvement in anthropometric parameters varied across trials, with some indicating significant improvements while others showed no significant changes. Reproductive outcomes primarily focused on menstrual cycle frequency and clinical and biochemical hyperandrogenism. Improvement in QoL was reported in seven trials, while three trials reported enhancement in mindfulness-related traits. Adverse events were infrequently reported, with some trials observing their absence while others reported incidents, such as kidney stone development and muscle cramping, albeit with uncertain causal relationships to the interventions.

## Discussion

The study provides a brief description of existing evidence concerning the efficacy of MMIs in alleviating symptoms associated with PCOS. The findings contribute to informing both ongoing and future research in this field. Most of the trials studied the impact of MMIs on psychological parameters, with a noteworthy absence of studies investigating pregnancy and related outcomes. A significant gap was found in the reporting of adherence and adverse events associated with MMIs. The current state of reporting regarding MMI delivery is deemed suboptimal, highlighting the necessity for improvement in future studies.

The concept and utilisation of meditation in the Eastern world transcends its role as a mere disease management tool, including aspects of self-help and spiritual well-being (52). Specific meditative techniques are seamlessly integrated into traditional health approaches in these countries. For example, meditation is included in yoga and Ayurveda (53). The diversity in the forms of MMIs used across various settings is evident. For instance, *yogic* meditation was the most used in India, while mindfulness-based practices were extensively explored in the USA. This discrepancy could be attributed to the cultural adaptation of meditation practices in Western contexts (54). Thus, it is vital to contextualise these practices when reporting or evaluating their effects. Furthermore, future studies should rigorously document and explain both the characteristics of the intervention provider and the intervention itself.

The implementation of complementary strategies, such as MMIs, holds promise, particularly in high-risk population groups, such as adolescents. This demographic stands to derive substantial benefits from complementary strategies aimed at facilitating weight management, improving lifestyle choices and mitigating the onset of reproductive-endocrine disorders (55). Meditation serves as a practical, cost-effective tool offering both physical and emotional benefits that extend beyond single interventions. Insights derived from studies covering adolescents and women afflicted with PCOS highlight the utility of meditation in facilitating psychological and metabolic outcomes amongst adolescent populations. These outcomes encompassed improvements in self-efficacy across various lifestyle domains. These findings are similar to observations from previous studies examining meditation and mindfulness interventions in non-PCOS adolescent populations, wherein significant reductions in psychological and emotional distress and improvements in self-esteem, mindfulness traits and overall quality of life (56–59) were observed. Furthermore, evidence supports the role of MMIs in mitigating addiction rates, increasing resilience and improving self-efficacy in managing chronic illnesses among this population (60–64). Consequently, early engagement in healthy lifestyle behaviours and MMIs among adolescent girls holds the potential to serve as a cornerstone in the management of PCOS. Future large studies with extended durations and employing appropriate diagnostic criteria among adolescents diagnosed with PCOS, and those at risk of developing PCOS, are warranted to explore the role of MMIs in PCOS management comprehensively.

Psychosocial co-morbidities are highly prevalent amongst women with PCOS and often contribute to reduced QoL (65). The majority of studies included in this review reported that MMIs reduced stress, anxiety and depression associated with PCOS. Similarly, the role of MMIs in improving psychological health, well-being and QoL was evident in non-PCOS individuals with mental health conditions (66–70). Golbery et al. concluded that meditation has results similar to standard treatments, such as psychotherapy for mental health conditions. A recent RCT by Bringmann et al. demonstrated that a meditation-based lifestyle program can help manage mild-moderate depression in the non-PCOS population (71). These findings indicate the potential of MMIs as a psychotherapeutic tool in managing psychological well-being, warranting further investigation in women with PCOS.

Lifestyle modifications are the primary therapeutic approach in the management of PCOS among women (1), primarily aiming at weight management through a combination of dietary modifications, exercise regimens and behavioural changes (72). However, women often encounter several barriers while adopting a healthy lifestyle (73, 74). A significant correlation between obesity, state of mind and food cravings aggravates this challenge, forming a vicious cycle (75). Previous studies involving non-PCOS adults support the effectiveness of MMIs not only in facilitating weight reduction (76, 77), but also in changing obesity-related eating behaviours, such as emotional, binge, and excess eating, thereby facilitating weight management (76, 78–83). Similarly, the study findings suggest that MMIs may improve self-efficacy and promote weight loss among individuals with PCOS (42, 43, 47, 48, 51).

Studies assessing the role of MMIs in eating behaviours suggested improvements in individuals’ relationships with food and reductions in psychological distress among those struggling with obesity (84). For example, mindful eating practices increase awareness of hunger, fullness, cravings, and triggers associated with dietary habits (85). MMIs also directly target ingrained behavioural patterns, such as faulty eating patterns, cravings, and addictions (86, 87). Thus, the exploration of MMIs is proposed as it holds promise in optimising lifestyle modifications and facilitating weight management among women afflicted with PCOS.

## Strengths and limitations

The adoption of the PRISMA-ScR checklist facilitated the identification of studies examining the utilisation of MMIs for managing PCOS. However, it is essential to acknowledge certain limitations that should be considered when interpreting the findings. All included studies were not critically appraised as this was a scoping review. The categorisation of MMIs proved challenging due to the complexity of interventions, which often comprised multifaceted practices, such as yoga therapy, unspecified meditation techniques, and instances where mindfulness overlapped with other forms of meditation. The present study did not aim to examine the efficacy of individual MMIs on specific PCOS features, as it was beyond the scope of the present study.

## Recommendations

This scoping review identified 14 studies investigating the breadth of MMIs for PCOS management. Several areas for future research have been identified to facilitate the translation of the findings presented in this review. These include (1): Future primary studies should explore the role of MMIs in promoting healthy lifestyle habits among adolescents (2); Future primary studies should examine the efficacy of MMIs on weight management and reproductive parameters in women diagnosed with PCOS (3); It is essential to establish clear definitions for the intervention, instructor and meditator, while also outlining proposed outcomes and implementing strategies to improve adherence rates; (4) Determination of therapeutic dosage levels of MMIs is needed to elicit clinically beneficial effects on PCOS features and describe the underlying mechanisms; (5) More rigorous methodologies for monitoring and documenting adverse events are required; (6) Further high-quality research, including attention-matched controls, is required to facilitate the integration of MMIs into clinical practice.

## Conclusions

PCOS is a common chronic condition accompanied by significant psycho-social burdens throughout individuals’ lifespans. The rising popularity and accumulating evidence of supporting holistic approaches, such as meditation and mindfulness, have demonstrated their benefits in improving psychological well-being and QoL outcomes. MMIs may serve as a valuable complement to standard PCOS care; however, their efficacy requires further exploration through high-quality research.

## Author contributions

VR: Conceptualization, Data curation, Formal Analysis, Investigation, Methodology, Project administration, Resources, Software, Validation, Writing – original draft, Writing – review & editing. AP: Methodology, Supervision, Validation, Writing – review & editing. AJ: Writing – review & editing, Data curation. AP: Data curation, Writing – review & editing, Formal Analysis. JG: Data curation, Writing – review & editing, Formal Analysis. EB: Writing – review & editing. NC: Writing – review & editing. PK: Funding acquisition, Investigation, Methodology, Supervision, Validation, Writing – review & editing.

## References

[1] Teede HJ Tay CT Laven JJE Dokras A Moran LJ Piltonen TT. Recommendations from the 2023 international evidence-based guideline for the assessment and management of polycystic ovary syndrom Eur J Endocrinology. (2023) 189:G43–64. doi: 10.1093/ejendo/lvad096 37580861

[2] Teede H Deeks A Moran L. Polycystic ovary syndrome: a complex condition with psychological, reproductive and metabolic manifestations that impacts on health across the lifespan. BMC Med. (2010) 8:41. doi: 10.1186/1741-7015-8-41 20591140 PMC2909929

[3] Gibson-Helm M Teede H Dunaif A AJTJoCE D. Metabolism. Delayed diagnosis and a lack of information associated with dissatisfaction in women with polycystic ovary syndrome. J Clin Endocrinol Metab. (2017) 102(2):604–12. doi: 10.1210/jc.2016-2963 PMC6283441 27906550

[4] Rao VS Cowan S Armour M Smith CA Cheema BS Moran L. A global survey of Ethnic Indian women living with polycystic ovary syndrome: Co-morbidities, concerns, diagnosis experiences, quality of life, and use of treatment methods. Int J Environ Res Public Health. (2022) 19:15850. doi: 10.3390/ijerph192315850 36497927 PMC9740300

[5] Peña AS Teede H Hewawasam E Hull ML Gibson-Helm M. Diagnosis experiences of adolescents with polycystic ovary syndrome: Cross-sectional study. Clin endocrinology. (2022) 96:62–9. doi: 10.1111/cen.14604 34585425

[6] Elghobashy M Lau GM Davitadze M Gillett CDT O’Reilly MW Arlt W. Concerns and expectations in women with polycystic ovary syndrome vary across age and ethnicity: findings from PCOS Pearls Study. Front Endocrinol (Lausanne). (2023) 14:1175548. doi: 10.3389/fendo.2023.1175548 37621648 PMC10446892

[7] Ee C Smith C Moran L MacMillan F Costello M Baylock B. “The whole package deal”: experiences of overweight/obese women living with polycystic ovary syndrome. BMC women’s Health. (2020) 20:1–9. doi: 10.1186/s12905-020-01090-7 33008386 PMC7532653

[8] Pirotta S Joham AE Moran LJ Skouteris H Lim SS. Implementation of the polycystic ovary syndrome guidelines: A mixed method study to inform the design and delivery of a lifestyle management program for women with polycystic ovary syndrome. Nutr Dietetics. (2021) 78:476–86. doi: 10.1111/1747-0080.12670 33876532

[9] Tay CT Pirotta S Teede HJ Moran LJ Robinson T Skouteris H. (2021). Polycystic ovary syndrome models of care: a review and qualitative evaluation of a guideline-recommended integrated care, in: Seminars in Reproductive Medicine, 333 Seventh Avenue, 18th Floor, NY, USA: Thieme Medical Publishers, Inc. 10.1055/s-0041-1727191 34187051

[10] Sheikh J Khalil H Shaikh S Hebbar M Zia N Wicks S. Emotional and psychosexual well-being is influenced by ethnicity and birthplace in women and individuals with polycystic ovary syndrome in the UK and India. BJOG: Int J Obstetrics Gynaecology. (2023) 130:978–86. doi: 10.1111/1471-0528.17428 PMC10952802 36807756

[11] Verma A Upadhyay V Saxena V. Effect of yoga therapy on health outcomes in women with polycystic ovary syndrome: A systematic review and meta-analysis. Am J lifestyle Med. (2023) 17:73–92. doi: 10.1177/15598276211029221 36636398 PMC9830238

[12] Rao VS Armour M Cheema BS Smith CA Moran L Perera RS. Use of traditional and complementary medicine by ethnic Indian women living with polycystic ovary syndrome: a global survey BMC Complementary Med Therapies. (2023) 23:392. doi: 10.1186/s12906-023-04229-9 PMC10623873 37924068

[13] Arentz S Smith CA Abbott JA Bensoussan A. A survey of the use of complementary medicine by a self-selected community group of Australian women with polycystic ovary syndrome BMC complementary and alternative. Medicine. (2014) 14:472. doi: 10.1186/1472-6882-14-472 PMC4265410 25481654

[14] Patañjali and Johnston, Charles. The Yoga sutras of Patanjali: the book of the spiritual man: an interpretation: Revised edition. Vol. 1975. London: Watkins. (1975). Available at: https://cir.nii.ac.jp/crid/1130000793975484416

[15] Cardoso R de Souza E Camano L Roberto Leite J. Meditation in health: an operational definition. Brain Res Protoc. (2004) 14:58–60. doi: 10.1016/j.brainresprot.2004.09.002 15519952

[16] Walsh R Shapiro SL. The meeting of meditative disciplines and Western psychology: a mutually enriching dialogue. Am Psychol. (2006) 61:227. doi: 10.1037/0003-066X.61.3.227 16594839

[17] Lutz A Dunne JD Davidson RJ. Meditation and the neuroscience of consciousness. In: Cambridge handbook of consciousness. Zelazo P.D. Moscovitch M. Thompson E. (Cambridge: Cambridge University Press), (2007). p. 499–555.

[18] Lutz A Slagter HA Dunne JD Davidson RJ. Attention regulation and monitoring in meditation. Trends Cogn Sci. (2008) 12:163–9. doi: 10.1016/j.tics.2008.01.005 PMC2693206 18329323

[19] Travis F Shear J. Focused attention, open monitoring and automatic self-transcending: Categories to organize meditations from Vedic, Buddhist and Chinese traditions. Consciousness Cognition. (2010) 19:1110–8. doi: 10.1016/j.concog.2010.01.007 20167507

[20] Kabat-Zinn J. Mindfulness-based interventions in context: past, present, and future. Clinical Psychology: Science and Practice (2003) 10(2):144–56. doi: 10.1093/clipsy/bpg016

[21] Baer RA Mindfulness training as a clinical intervention: A conceptual and empirical review Clin psychology: Sci practice (2003) 10:125. doi: 10.1093/clipsy/bpg015

[22] Garland EL Hanley AW Riquino MR Reese SE Baker AK Salas K. Mindfulness-oriented recovery enhancement reduces opioid misuse risk via analgesic and positive psychological mechanisms: A randomized controlled trial. J Consulting Clin Psychol. (2019) 87:927. doi: 10.1037/ccp0000390 PMC6764586 31556669

[23] Garland EL Baker AK Riquino MR Priddy SE. Mindfulness-oriented recovery enhancement. Handb mindfulness-based program mindfulness. Interv Educ to heal Ther Abingdon. (2019) 13:327–40.

[24] Kabat-Zinn J Lipworth L Burney R. The clinical use of mindfulness meditation for the self-regulation of chronic pain. J Behav Med. (1985) 8:163–90. doi: 10.1007/BF00845519 3897551

[25] White ND. Mindfulness-based cognitive therapy for depression, current episodes, and prevention of relapse. Am J Lifestyle Med. (2015) 9:227–9. doi: 10.1177/1559827615569677

[26] Van Dam NT Van Vugt MK Vago DR Schmalzl L Saron CD Olendzki A. Mind the hype: A critical evaluation and prescriptive agenda for research on mindfulness and meditation. Perspect psychol science. (2018) 13:36–61. doi: 10.1177/1745691617709589 PMC5758421 29016274

[27] Orme-Johnson D. Medical care utilization and the transcendental meditation program Psychosomatic Med. (1987) 49(5):493–50. doi: 10.1097/00006842-198709000-00006 3313489

[28] Shapiro SL Carlson LE Astin JA Freedman B. Mechanisms of mindfulness. J Clin Psychol. (2006) 62:373–86. doi: 10.1002/jclp.20237 16385481

[29] Schlechta Portella CF Ghelman R Abdala V Schveitzer MC Afonso RF. Meditation: evidence map of systematic reviews. Front Public Health. (2021) 9:1777. doi: 10.3389/fpubh.2021.742715 PMC8674467 34926371

[30] Bursky M Kosuri M Walsh Carson K Babad S Iskhakova A Nikulina V. The utility of meditation and mindfulness-based interventions in the time of COVID-19: A theoretical proposition and systematic review of the relevant prison, quarantine and lockdown literature. psychol Rep. (2023) 126:557–600. doi: 10.1177/00332941211048734 34889700 PMC10037136

[31] Phimphasone-Brady P Palmer B Vela A Johnson RL Harnke B Hoffecker L. Psychosocial interventions for women with polycystic ovary syndrome: a systematic review of randomized controlled trials. F&S Rev. (2022) 3:42–56. doi: 10.1016/j.xfnr.2021.11.004

[32] Reuters T. Thomson reuters Int J Court Admin. (2014) 6(2).

[33] Covidence systematic review software. Veritas Health Innovation. Melbourne, Australia (2013). Available at: https://www.covidence.org (accessed 15 April 2023)

[34] Nidhi R Padmalatha V Nagarathna R Amritanshu R. Effects of a holistic yoga program on endocrine parameters in adolescents with polycystic ovarian syndrome: A randomized controlled trial. J Altern Complementary Med. (2012) 19:153–60. doi: 10.1089/acm.2011.0868 22808940

[35] Nidhi R Padmalatha V Nagarathna R Amritanshu R. Effect of yoga program on quality of life in adolescent polycystic ovarian syndrome: A randomized control trial. Appl Res Qual Life. (2013) 8:373–83. doi: 10.1007/s11482-012-9191-9

[36] Nidhi R Padmalatha V Nagarathna R Amritanshu R. Effect of holistic yoga program on anxiety symptoms in adolescent girls with polycystic ovarian syndrome: A randomized control trial. Int J yoga. (2012) 5:112–7. doi: 10.4103/0973-6131.98223 PMC3410189 22869994

[37] Nidhi R Padmalatha V Nagarathna R Ram A. Effect of a yoga program on glucose metabolism and blood lipid levels in adolescent girls with polycystic ovary syndrome. Int J gynaecology obstetrics: Off Organ Int Fed Gynaecology Obstetrics. (2012) 118:37–41. doi: 10.1016/j.ijgo.2012.01.027 22507264

[38] Stefanaki C Bacopoulou F Livadas S Kandaraki A Karachalios A Chrousos GP. Impact of a mindfulness stress management program on stress, anxiety, depression and quality of life in women with polycystic ovary syndrome: a randomized controlled trial. Stress (Amsterdam Netherlands). (2015) 18:57–66. doi: 10.3109/10253890.2014.974030 25287137

[39] Vibhuti Rao B Kashinath Metri B Shubhashankari Rao B Nagaratna R. Improvement in biochemical and psychopathologies in women having PCOS through yoga combined with herbal detoxification. J Stem Cells. (2018) 13:213–22.

[40] Patel V Menezes H Menezes C Bouwer S Bostick-Smith CA Speelman DL. Regular mindful yoga practice as a method to improve androgen levels in women with polycystic ovary syndrome: A randomized, controlled trial. J Am Osteopathic Assoc. (2020) 120(5):323–35. doi: 10.7556/jaoa.2020.050 32285088

[41] Yin MXC Du LB Zou XN Fung YL Sun YY Chan CHY. Can psychosocial intervention suppress testosterone and triglycerides among women with polycystic ovary syndrome? A feasibility trial. Front Psychol. (2021) 12:690539. doi: 10.3389/fpsyg.2021.690539 34367014 PMC8339270

[42] Young CC Monge M Minami H Rew L Conroy H Peretz C. Outcomes of a mindfulness-based healthy lifestyle intervention for adolescents and young adults with polycystic ovary syndrome. J Pediatr Adolesc gynecology. (2022) 35:305–13. doi: 10.1016/j.jpag.2021.10.016 PMC9065214 34742935

[43] Dema H Videtič Paska A Kouter K Katrašnik M Jensterle M Janež A. Effects of mindfulness-based therapy on clinical symptoms and DNA methylation in patients with Polycystic ovary syndrome and high metabolic risk. Curr Issues Mol Biol. (2023) 45:2717–37. doi: 10.3390/cimb45040178 PMC10136994 37185702

[44] Jakhar R Sen ED Rastogi P. Improvement in health-related quality of life in polycystic ovarian syndrome: A randomized controlled trial. J Survey Fisheries Sci. (2023), 382–8. doi: 10.17762/sfs.v10i1.558

[45] Raja-Khan N Agito K Shah J Stetter CM Gustafson TS Socolow H. Mindfulness-based stress reduction in women with overweight or obesity: a randomized clinical trial. Obesity. (2017) 25:1349–59. doi: 10.1002/oby.21910 PMC5529243 28686006

[46] Vanitha A Pandiaraja M Maheshkumar K Venkateswaran S. Effect of yoga nidra on resting cardiovascular parameters in polycystic ovarian syndrome women. Natl J Physiology Pharm Pharmacol. (2018) 8:1505–8. doi: 10.5455/njppp.

[47] Verma A Gandhi A Gautam S Biswas R Mondal S. Effect of yogic techniques on Heart Rate Variability in Polycystic Ovarian Syndrome patients. Journal of Dental and Medical Sciences (2019) 8(2):28–34. doi: 10.9790/0853-1803102834

[48] Missel AL O’Brien AV Maser H Kanwal A Bayandorian H Martin S. Impact of an online multicomponent very-low-carbohydrate program in women with polycystic ovary syndrome: a pilot study F&S Rep. (2021) 2:386–95. doi: 10.1016/j.xfre.2021.08.008 PMC8655401 34934978

[49] Sode J Bhardwaj MA. Effect of Yoga on Level of Depression among Females suffering from Polycystic Ovarian Syndrome (PCOS). Int J Arts Manag Humanit (2017) 6(2):178–81. Available at: https://pdfs.semanticscholar.org/7154/c114c4eccbf04f2a0444b88b789c1423b77d.pdf.

[50] Salajegheh Z Ahmadi A Shahrahmani H Jahani Y Alidousti K Nasiri Amiri F. Mindfulness-based stress reduction (MBSR) effects on the worries of women with poly cystic ovary syndrome (PCOS). BMC Psychiatry. (2023) 23:185. doi: 10.1186/s12888-023-04671-6 36944940 PMC10032018

[51] Raja-Khan N Agito K Shah J Stetter CM Gustafson TS Socolow H. Mindfulness-based stress reduction for overweight/obese women with and without polycystic ovary syndrome: design and methods of a pilot randomized controlled trial. Contemp Clin trials. (2015) 41:287–97. doi: 10.1016/j.cct.2015.01.021 PMC4380576 25662105

[52] Singla R. Origins of mindfulness & meditation interplayof eastern & western psychology. Psyke Logos. (2011) 32:20.

[53] Jaiswal YS Williams LL. A glimpse of Ayurveda–The forgotten history and principles of Indian traditional medicine J traditional complementary Med. (2017) 7:50–3. doi: 10.1016/j.jtcme.2016.02.002 PMC5198827 28053888

[54] Crosby K. Esoteric Theravada: the story of the forgotten meditation tradition of Southeast Asia. (Boulder, USA: Shambhala Publications) (2020).

[55] Dobbie LJ Pittam B Zhao SS Alam U Hydes TJ Barber TM. Childhood, adolescent, and adulthood adiposity are associated with risk of PCOS: a Mendelian randomization study with meta-analysis. Hum Reproduction. (2023) 38:1168–82. doi: 10.1093/humrep/dead053 PMC10233304 37015099

[56] Shomaker LB Bruggink S Pivarunas B Skoranski A Foss J Chaffin E. Pilot randomized controlled trial of a mindfulness-based group intervention in adolescent girls at risk for type 2 diabetes with depressive symptoms. Complementary Therapies Med. (2017) 32:66–74. doi: 10.1016/j.ctim.2017.04.003 PMC5705100 28619307

[57] Shomaker LB Pivarunas B Annameier SK Gulley L Quaglia J Brown KW. One-year follow-up of a randomized controlled trial piloting a mindfulness-based group intervention for adolescent insulin resistance Front Psychol. (2019) 10. doi: 10.3389/fpsyg.2019.01040 PMC6517501 31133946

[58] Tan L Martin G. Taming the adolescent mind: a randomised controlled trial examining clinical efficacy of an adolescent mindfulness-based group programme. Child Adolesc Ment Health. (2015) 20:49–55. doi: 10.1111/camh.12057 32680328

[59] Black DS Milam J Sussman S. Sitting-meditation interventions among youth: A review of treatment efficacy. Pediatrics. (2009) 124:e532–e41. doi: 10.1542/peds.2008-3434 PMC3195513 19706568

[60] Ahola Kohut S Stinson J Davies-Chalmers C Ruskin D van Wyk M. Mindfulness-based interventions in clinical samples of adolescents with chronic illness: A systematic review. J Altern Complementary Med. (2017) 23:581–9. doi: 10.1089/acm.2016.0316 28355082

[61] Young CC Rew L Monge M. Transition to self-management among adolescents with polycystic ovary syndrome: parent and adolescent perspectives. J Pediatr nursing. (2019) 47:85–91. doi: 10.1016/j.pedn.2019.04.024 PMC6642853 31079016

[62] Raja R. A study of vipassana meditation on adolescent behaviour pattern. Inst for Yoga & Consciousness (2005) India: 23(1):12–24, 0379-3885(Print).

[63] Tan LB. A critical review of adolescent mindfulness-based programmes. Clin Child Psychol Psychiatry. (2016) 21:193–207. doi: 10.1177/1359104515577486 25810416

[64] Khanna S Greeson JM. A narrative review of yoga and mindfulness as complementary therapies for addiction. Complementary therapies Med. (2013) 21:244–52. doi: 10.1016/j.ctim.2013.01.008 PMC3646290 23642957

[65] Dokras A Stener-Victorin E Yildiz BO Li R Ottey S Shah D. Androgen Excess- Polycystic Ovary Syndrome Society: position statement on depression, anxiety, quality of life, and eating disorders in polycystic ovary syndrome. Fertility Sterility. (2018) 109:888–99. doi: 10.1016/j.fertnstert.2018.01.038 29778388

[66] Álvarez-Pérez Y Rivero-Santana A Perestelo-Pérez L Duarte-Díaz A Ramos-García V Toledo-Chávarri A. Effectiveness of mantra-based meditation on mental health: A systematic review and meta-analysis. Int J Environ Res Public Health. (2022) 19:3380. doi: 10.3390/ijerph19063380 35329068 PMC8949812

[67] Hendriks T. The effects of Sahaja Yoga meditation on mental health: a systematic review. J Complementary Integr Med. (2018) 15:20160163. doi: 10.1515/jcim-2016-0163 29847314

[68] Rainforth MV Schneider RH Nidich SI Gaylord-King C Salerno JW Anderson JW. Stress reduction programs in patients with elevated blood pressure: a systematic review and meta-analysis. Curr Hypertens Rep. (2007) 9:520–8. doi: 10.1007/s11906-007-0094-3 PMC2268875 18350109

[69] Goldberg SB Tucker RP Greene PA Davidson RJ Wampold BE Kearney DJ. Mindfulness-based interventions for psychiatric disorders: A systematic review and meta-analysis. Clin Psychol review. (2018) 59:52–60. doi: 10.1016/j.cpr.2017.10.011 PMC5741505 29126747

[70] Vancampfort D Stubbs B Van Damme T Smith L Hallgren M Schuch F. The efficacy of meditation-based mind-body interventions for mental disorders: A meta-review of 17 meta-analyses of randomized controlled trials. J Psychiatr Res. (2021) 134:181–91. doi: 10.1016/j.jpsychires.2020.12.048 33388701

[71] Bringmann HC Michalsen A Jeitler M Kessler CS Brinkhaus B Brunnhuber S. Meditation-based lifestyle modification in mild to moderate depression—A randomized controlled trial. Depression Anxiety. (2022) 39:363–75. doi: 10.1002/da.23249 35312137

[72] Lim SS Hutchison SK Van Ryswyk E Norman RJ Teede HJ Moran LJ. Lifestyle changes in women with polycystic ovary syndrome Cochrane Database Systematic Rev. (2019) 3(3):CD007506. doi: 10.1002/14651858.CD007506.pub4 PMC6438659 30921477

[73] Blackshaw LC Chhour I Stepto NK Lim SS. Barriers and facilitators to the implementation of evidence-based lifestyle management in polycystic ovary syndrome: a narrative review. Med Sci. (2019) 7:76. doi: 10.3390/medsci7070076 PMC6681274 31252682

[74] Ee C Pirotta S Mousa A Moran L Lim S. Providing lifestyle advice to women with PCOS: an overview of practical issues affecting success. BMC endocrine Disord. (2021) 21:1–12. doi: 10.1186/s12902-021-00890-8 PMC8609880 34814919

[75] Stefanaki K Karagiannakis DS Raftopoulou M Psaltopoulou T Paschou SA Ilias I. Obesity and hyperandrogenism are implicated with anxiety, depression and food cravings in women with polycystic ovary syndrome. Endocrine. (2023) 82:201–8. doi: 10.21203/rs.3.rs-2513408/v1 37389719

[76] Carrière K Khoury B Günak MM Knäuper B. Mindfulness-based interventions for weight loss: a systematic review and meta-analysis. Obes Rev. (2018) 19:164–77. doi: 10.1111/obr.12623 29076610

[77] Rogers JM Ferrari M Mosely K Lang CP Brennan L. Mindfulness-based interventions for adults who are overweight or obese: a meta-analysis of physical and psychological health outcomes. Obes Rev. (2017) 18:51–67. doi: 10.1111/obr.12461 27862826

[78] O’Reilly GA Cook L Spruijt-Metz D Black D. Mindfulness-based interventions for obesity-related eating behaviours: a literature review. Obes Rev. (2014) 15:453–61. doi: 10.1111/obr.12156 PMC4046117 24636206

[79] Olson KL Emery CF. Mindfulness and weight loss: A systematic review. Psychosomatic Med. (2015) 77:59–67. doi: 10.1097/PSY.0000000000000127 25490697

[80] Dalen J Smith BW Shelley BM Sloan AL Leahigh L Begay D. Pilot study: Mindful Eating and Living (MEAL): weight, eating behavior, and psychological outcomes associated with a mindfulness-based intervention for people with obesity. Complementary therapies Med. (2010) 18:260–4. doi: 10.1016/j.ctim.2010.09.008 21130363

[81] Barber TM Hanson P Weickert MO Franks S. Obesity and polycystic ovary syndrome: implications for pathogenesis and novel management strategies. Clin Med Insights: Reprod Health. (2019) 13:1179558119874042. doi: 10.1177/1179558119874042 31523137 PMC6734597

[82] Hanson P Shuttlewood E Halder L Shah N Lam F Menon V. Application of mindfulness in a tier 3 obesity service improves eating behavior and facilitates successful weight loss. J Clin Endocrinol Metab. (2019) 104:793–800. doi: 10.1210/jc.2018-00578 30566609

[83] Fuentes Artiles R Staub K Aldakak L Eppenberger P Rühli F Bender N. Mindful eating and common diet programs lower body weight similarly: Systematic review and meta-analysis. Obes Rev. (2019) 20:1619–27. doi: 10.1111/obr.12918 31368631

[84] Mantzios M Wilson JC. Mindfulness, eating behaviours, and obesity: a review and reflection on current findings. Curr Obes Rep. (2015) 4:141–6. doi: 10.1007/s13679-014-0131-x 26627097

[85] Monroe JT. Mindful eating: principles and practice. Am J Lifestyle Med. (2015) 9:217–20. doi: 10.1177/1559827615569682

[86] Kristeller JL. Mindfulness, eating disorders, and food intake regulationIn: Handbook of mindfulness and self-regulation. (2015) (New York Heidelberg Dordrecht London: Springer). p. 199–215.

[87] Vadivale AM Sathiyaseelan A. Mindfulness-based relapse prevention–A meta-analysis. Cogent Psychol. (2019) 6:1567090. doi: 10.1080/23311908.2019.1567090

