# Supplementary material for: The role of meditation and mindfulness in the management of polycystic ovary syndrome: a scoping review
Source: Front Endocrinol (Lausanne). 2024 May 16;15:1295705. doi: 10.3389/fendo.2024.1295705 (PMC11137171; doi:10.3389/fendo.2024.1295705)
Supplement: Supplementary file 1 [file Table_1.docx]

Supplementary Material

**Supplemental Table 1: Ovid Medline Search Strategy**

Ovid MEDLINE (R) ALL (on 10^th^ March 2023)

| Search Terms | Outcome |
| --- | --- |
| Polycystic Ovary Syndrome.mp. or exp Polycystic Ovary Syndrome/ | 21757 |
| Polycystic Ovar$.tw. | 20935 |
| (PCOS or PCOD).tw. | 15138 |
| (sclerocystic adj3 ovar$).tw. | 105 |
| stein leventhal.tw. | 619 |
| 1 or 2 or 3 or 4 or 5 | 24389 |
| Meditation.mp. or Meditation/ | 7955 |
| Mind-Body Therapies.mp. or Mind-Body Therapies/ | 1503 |
| Mindfulness.mp. or Mindfulness/ or Meditation/ | 15204 |
| (mind-body adj2 (therap* or program* or medicin*)).tw. | 879 |
| meditation.tw. | 6329 |
| (MBSR or MBCT).tw. | 1443 |
| Meditation/ or Yoga/ or meditat*.mp. or Mindfulness/ | 16667 |
| Mindfulness/ or Mindful*.mp. | 16426 |
| Yoga.mp. or Yoga/ | 7067 |
| dhyan*.mp. or Yoga/ | 3777 |
| Yoga/ or yog*.mp. | 14447 |
| yoga.tw. | 6370 |
| 7 or 8 or 9 or 10 or 11 or 12 or 13 or 14 or 15 or 16 or 17 or 18 | 35924 |
| 6 and 19 | 33 |
